# Case Report: A case of severe chronic infection of an intraperitoneally placed composite mesh treated with complete mesh explantation and primary tissue repair using the anterior component separation technique

**DOI:** 10.3389/fsurg.2026.1892681

**Published:** 2026-10-02

**Authors:** Branko Bakula, Marko Sever, Ante Ivkošić, Zvonko Zadro, Maja Bakula, Ivan Romić, Davor Mijatović

**Affiliations:** 1 Department of Surgery, University Hospital Sveti Duh, Zagreb, Croatia; 2 School of Medicine, University of Zagreb, Zagreb, Croatia; 3 Department of Ophthalmology, University Hospital Centre Zagreb, Zagreb, Croatia; 4 Department of Surgery, University Hospital Centre Zagreb, Zagreb, Croatia

**Keywords:** anterior component separation, composite mesh, mesh infection, recurrent ventral hernia, ventral hernia repair

## Abstract

**Introduction:**

The introduction of synthetic mesh has fundamentally changed the management of ventral hernias by substantially reducing recurrence rates compared with primary suture repair. However, mesh implantation carries an inherent risk of foreign body–related complications, most notably mesh infection. Although uncommon, mesh infection following ventral hernia repair is a potentially devastating complication that often requires complex surgical management. Treatment strategies range from attempts at mesh preservation to complete mesh explantation followed by abdominal wall reconstruction with or without the use of a biologic mesh. The leading risk factors for mesh infection include postoperative wound complications and inappropriate mesh selection or placement.

**Case presentation:**

We present the case of a 29-year-old man who sustained a splenic rupture in a traffic accident. At another institution, he underwent emergency splenectomy, during which a large composite mesh was implanted in the intraperitoneal position. Approximately three months later, purulent discharge developed from the laparotomy wound, followed shortly thereafter by exposure of part of the mesh. He presented to our institution with a large ventral hernia, extensive exposure of an infected mesh, and persistent purulent drainage. Abdominal MSCT excluded an enteric fistula and demonstrated a ventral hernia with a transverse defect measuring 14 cm, together with conglomerates of contracted mesh located beneath the skin. Complete mesh explantation was performed, followed by definitive abdominal wall reconstruction without implantation of a new mesh. Primary tissue repair was achieved using anterior component separation to facilitate tension-free midline fascial closure. The operation and postoperative recovery were uneventful.

**Discussion:**

Mesh infection remains an important complication of ventral hernia repair and is likely to continue to be encountered in clinical practice. Nevertheless, the clinical benefits of mesh-based repair generally outweigh the associated risks, and mesh implantation remains the standard of care for most ventral hernias. Future efforts should therefore focus on preventing mesh infection, particularly through structured surgeon education on appropriate mesh selection, optimal mesh placement, and adherence to sound surgical principles in ventral hernia repair.

## Introduction

The introduction of synthetic mesh has fundamentally changed the management of ventral hernias. Compared with primary suture repair, mesh reinforcement has substantially reduced hernia recurrence rates and has become the standard of care for most abdominal wall reconstructions. However, these improvements were not achieved immediately after the introduction of mesh, as the optimal plane for mesh implantation remained a matter of ongoing investigation for many years. Accumulating clinical evidence has since established the sublay (retromuscular/preperitoneal) plane as the preferred site for mesh placement because of its favorable clinical outcomes (1).

Despite its excellent clinical outcomes, mesh implantation is associated with certain risks inherent to the placement of a foreign material, most notably mesh infection and, less frequently, mesh rejection. Nevertheless, the marked reduction in hernia recurrence achieved with mesh-based repair has established its widespread use, as its clinical benefits generally outweigh the associated risks (2).

Mesh infection is an uncommon but potentially serious complication of ventral hernia repair. Although its reported incidence ranges from 1% to 8%, it often presents a considerable therapeutic challenge and may require complex abdominal wall reconstruction (3, 4). Owing to the limited high-quality evidence, no universally accepted treatment guidelines currently exist. Management should therefore be individualized according to the extent and severity of infection, patient-related factors, and the characteristics of the implanted mesh. Treatment strategies range from complete mesh explantation to partial mesh removal or, in carefully selected cases, mesh preservation. Following mesh explantation, abdominal wall reconstruction may be performed using biological mesh or autologous tissue repair, depending on the clinical circumstances and the surgeon's preferred reconstructive strategy (5).

This report presents the case of a patient with a large ventral hernia complicated by chronic severe infection of an exposed composite mesh, successfully managed by complete mesh explantation followed by definitive abdominal wall reconstruction using primary tissue repair with anterior component separation.

## Case presentation

We present the case of a 29-year-old male patient with a body mass index (BMI) of 25 kg/m^2^ who was an active smoker and had no significant medical comorbidities. Eleven months before presenting to our institution, the patient sustained a splenic rupture in a motorcycle accident. Emergency splenectomy was performed at another institution through a midline laparotomy, during which a 30 × 30 cm composite mesh was implanted in the intraperitoneal (underlay) position. The rationale for mesh implantation during the emergency procedure was not documented in the available medical records.

Approximately three months after the initial emergency surgery, purulent discharge developed from the laparotomy wound, followed shortly thereafter by exposure of a portion of the mesh. According to the available medical records from another institution, the wound was initially managed conservatively for approximately six months with regular wound dressing changes, antibiotic therapy, and occasional removal of small fragments of exposed mesh during dressing changes.

The patient presented to our institution with a large ventral hernia, extensive exposure of an infected mesh, and persistent foul-smelling purulent drainage (Figures 1A,B). Despite these local findings, inflammatory parameters were within the normal range, and the patient was in good general condition. An abdominal MSCT with oral contrast administration excluded an enteric fistula and demonstrated a ventral hernia with a transverse defect measuring 14 cm, together with conglomerates of contracted mesh located immediately beneath the skin (Figure 1C).

**Figure 1:**
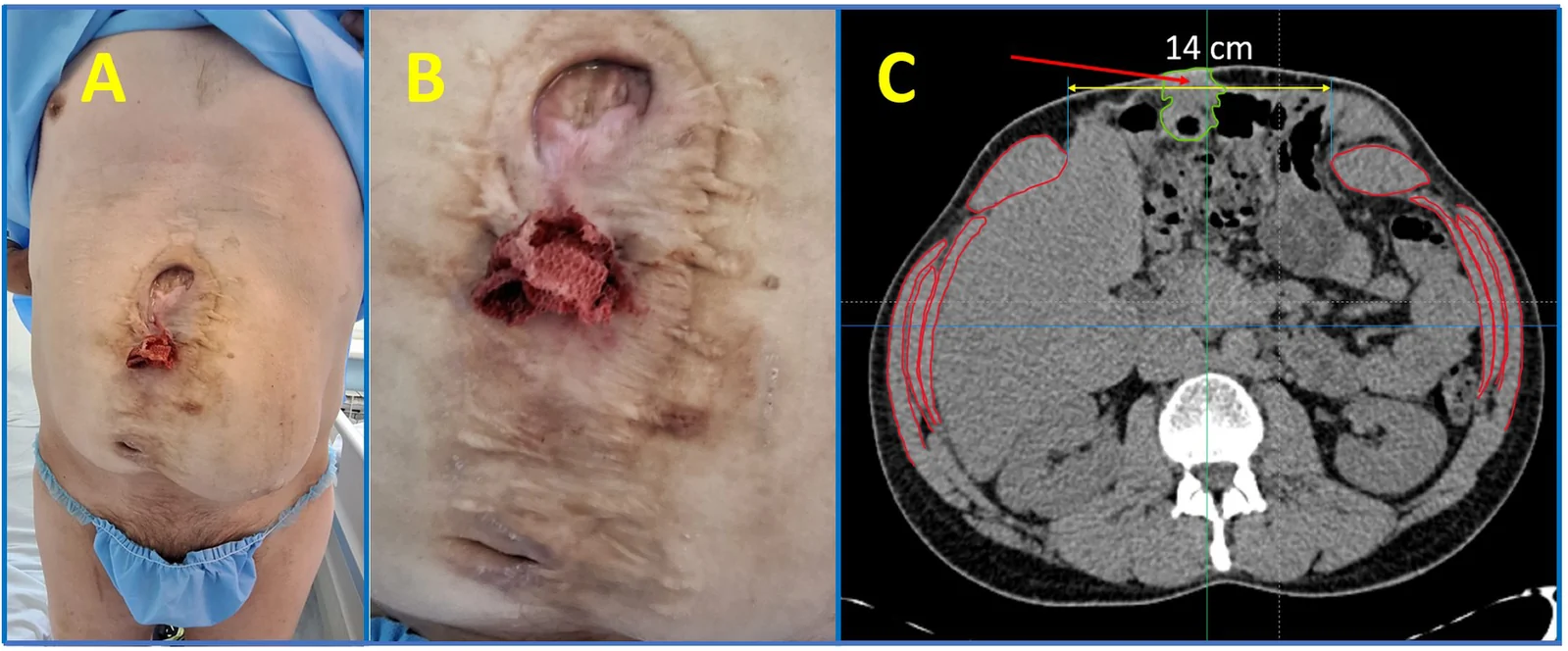
**(A)** A large ventral hernia with exposed mesh is seen in the standing position. **(B)** Preoperative view of exposed infected mesh with numerous purulent fistula openings on chronically macerated skin. **(C)** MSCT findings show a hernial defect 14 cm wide with a conglomerate of shrunken mesh under the skin (red arrow).

Prior to surgery, a wound swab was obtained for microbiological analysis. Polymicrobial colonization, including *Staphylococcus aureus*, *Escherichia coli*, and *Staphylococcus epidermidis*, was identified. Targeted intravenous antibiotic therapy with amoxicillin–clavulanate (1.2 g three times daily) and metronidazole (500 mg three times daily), based on the culture and susceptibility results, was initiated three days before surgery.

The procedure began with a wide elliptical excision of the scar, together with the chronically macerated skin and all devitalized tissue. The entire mesh was removed en bloc along with the overlying soft tissues. The mesh was markedly contracted and, fortunately, was not significantly adherent to the underlying bowel (Figure 2). The portion of segment III of the liver that had become adherent to and grown into the mesh was resected by performing a non-anatomical partial resection of segment III. As the resection was limited in extent, electrocautery alone was sufficient for parenchymal transection, resulting in minimal blood loss without any adverse effect on postoperative liver function. Histopathological examination revealed normal hepatic parenchyma with chronic inflammatory changes in the areas adherent to the mesh (Figure 3A).

**Figure 2:**
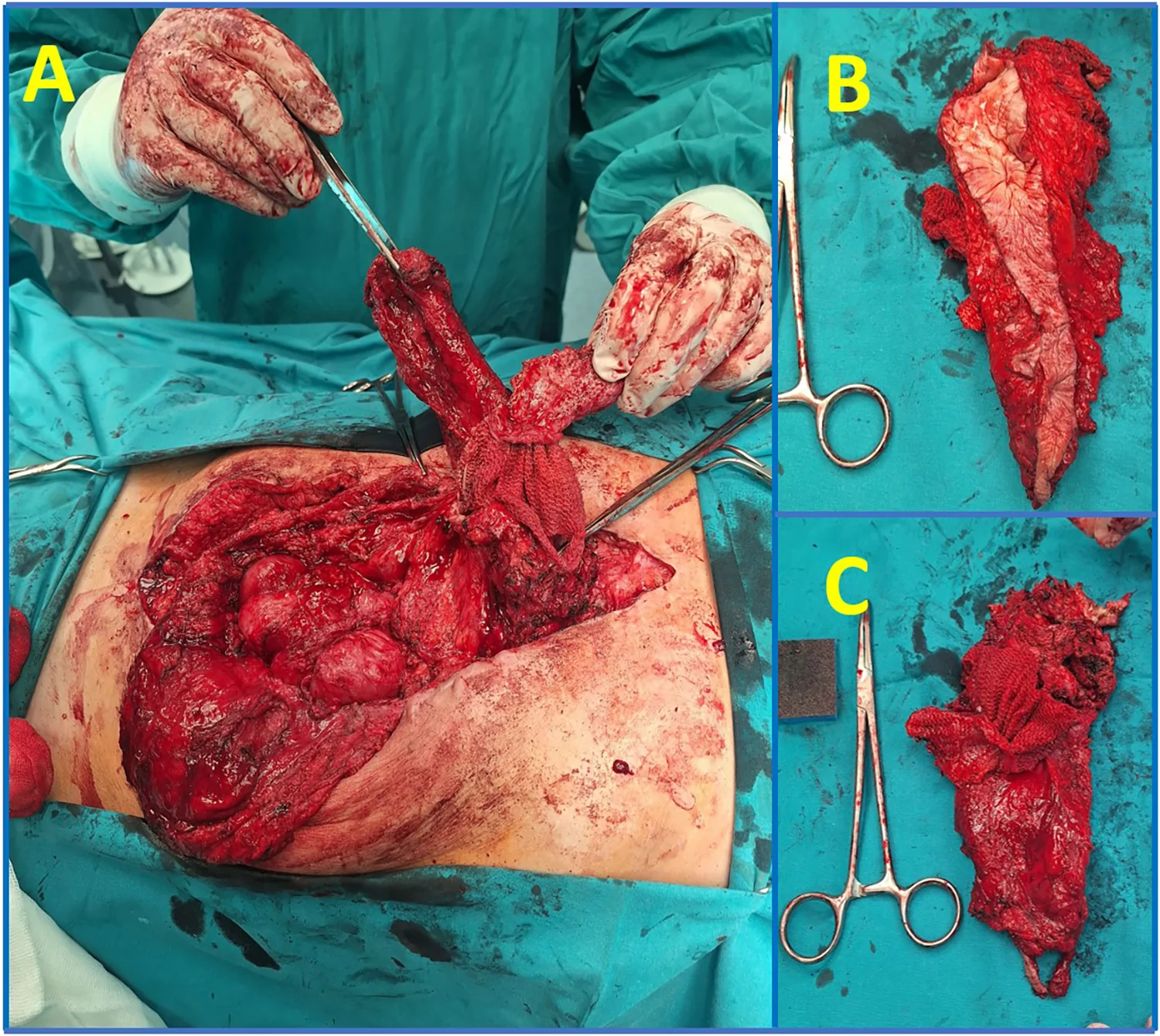
**(A)** Intraoperative image after dissection of the infected mesh. **(B)** and **(C)** Anterior and posterior view of the en bloc removed infected mesh along with the skin.

**Figure 3:**
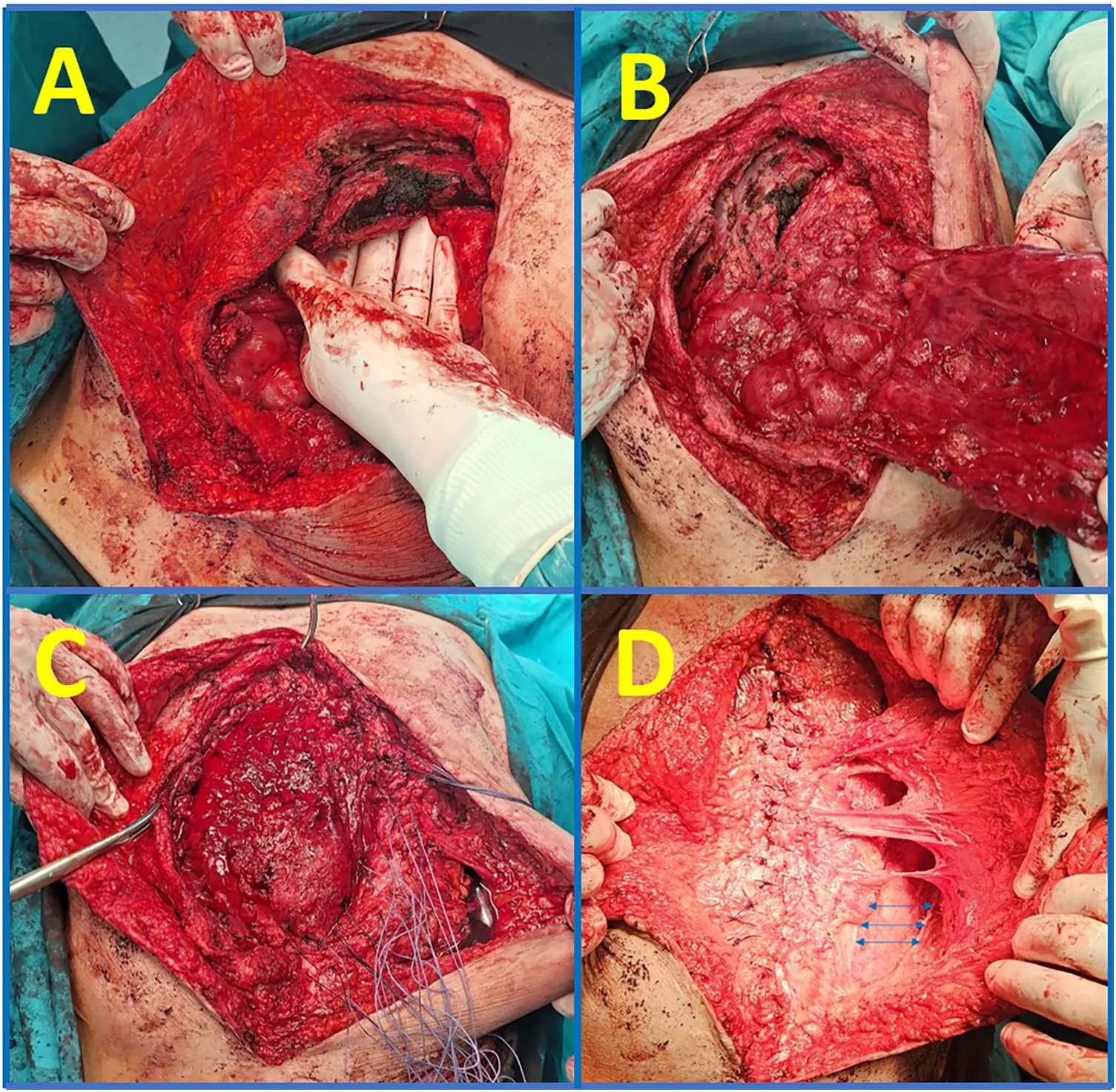
**(A)** Intraoperative view of the abdominal cavity after adhesiolysis and complete mesh removal. A partially resected third segment of the liver is seen. **(B)** The large hernial sac is fully mobilized and preserved. **(C)** The hernia sac is sutured to the contralateral side, thus serving as the first layer of abdominal closure. **(D)** Intraoperative view of abdominal cavity completely closed in the midline after unilateral anterior component separation performed with preserved some of the perforators. Blue arrows show the edges of the longitudinally incised aponeurosis of the external oblique muscle.

Furthermore, intra-abdominal adhesiolysis was performed and the healthy edges of the hernial defect were dissected while preserving the entire hernial sac, which will be used for later abdominal reconstruction (Figures 3B,C). As it was observed that the abdominal wall defect was too wide to be closed primarily, it was decided to initially perform a unilateral ACS according to Ramirez on the left side, where the subcutaneous space was more significantly mobilized due to the long-standing hernia. A generous longitudinal incision was made in the external oblique aponeurosis along the lateral border of the left rectus abdominis muscle, extending from the inguinal ligament to the costal margin (6). This maneuver resulted in approximately 8 cm of medial mobilization of the left rectus abdominis muscle. A traction test confirmed that the fascial edges could be approximated without tension, eliminating the need for contralateral component separation. During the dissection, effort was made to preserve the perforator vessels using the Saulis technique by maintaining a bridge of periumbilical subcutaneous tissue. However, complete preservation was not possible because the integrity of the subcutaneous tissue had already been significantly compromised by the large ventral hernia (Figure 3D).

Before definitive fascial closure, the hernia sac (peritoneum) was used to bridge and reinforce the hernia defect (7). Fascial closure was then achieved using interrupted sutures without significant tension. The extensive subcutaneous dead space was drained with two 19-Fr suction drains and further obliterated by meticulously suturing the skin flaps to the underlying fascia. Following additional excision of the remaining devitalized skin, the wound was closed with interrupted skin sutures (Figure 4A).

**Figure 4:**
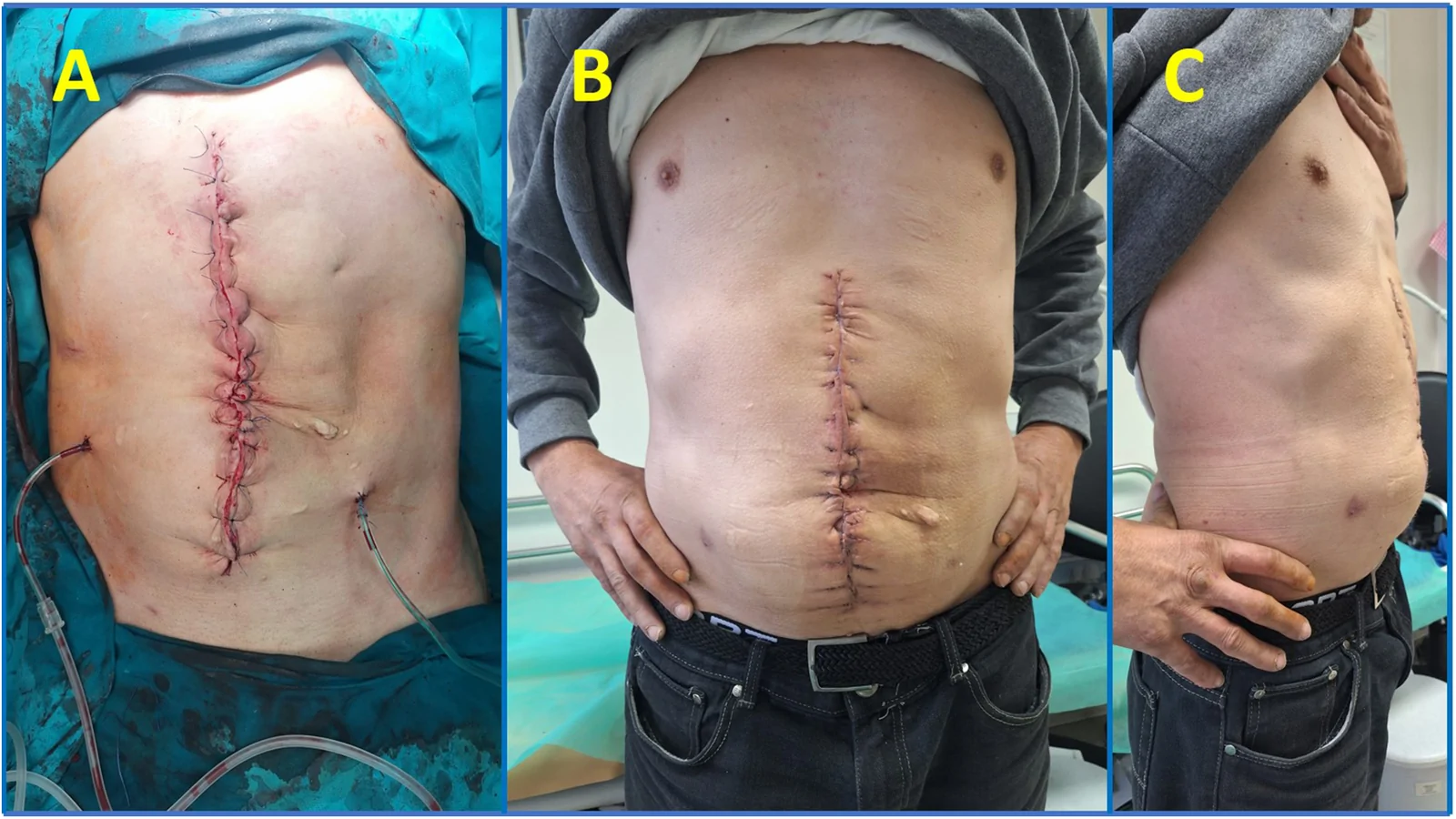
**(A)** View of the abdomen at the end of the operation. Two suction drains were used. **(B)** and **(C)** Postoperative view of the patient showing a fully functional dynamic abdominal wall.

The operation and early postoperative course were uneventful. Surgical drains were removed on postoperative days 5 and 6 after the daily serous output had decreased to less than 20 mL. The patient was discharged on postoperative day 8. At the most recent follow-up, 16 months after surgery, there were no signs of hernia recurrence, and the abdominal wall demonstrated full functional integrity and dynamic activity (Figures 4B,C).

## Discussion

Mesh infection is an uncommon but potentially devastating complication of ventral hernia repair. Large population-based studies suggest that mesh explantation after elective ventral hernia repair is relatively infrequent; however, the risk increases substantially in patients who develop early postoperative wound complications. Ehlers et al., in a retrospective analysis of 59,453 patients undergoing elective open ventral hernia repair with mesh, found that mesh explantation was required in only 2.2% of patients, but postoperative wound complications were strongly associated with subsequent mesh infection and explantation (8). These findings emphasize that early wound morbidity is an important predictor of later mesh-related infectious complications. Other recognized risk factors include concomitant bowel resection, inadvertent bowel injury, inappropriate mesh placement, bridging repair, emergency surgery, obesity, smoking, diabetes mellitus, and immunosuppressive therapy (9).

The clinical presentation of mesh infection is highly variable and may range from localized erythema and swelling to chronic purulent drainage, enterocutaneous fistula formation, mesh exposure, and recurrent hernia. The presence of a recurrent abdominal wall defect further complicates treatment by combining infection control with the need for subsequent abdominal wall reconstruction. Mesh infections are usually polymicrobial, with *Staphylococcus aureus* being the most frequently isolated microorganism, followed by Enterobacteriaceae and anaerobic bacteria, while methicillin-resistant *Staphylococcus aureus* (MRSA) has also become increasingly relevant (10).

Although conservative treatment may be successful in carefully selected patients with limited mesh infection, its effectiveness is generally restricted to specific clinical situations. Prolonged antibiotic therapy, drainage, local wound care, and negative-pressure wound therapy (VAC) may allow mesh preservation in selected cases; however, established or extensive infection frequently requires surgical treatment and, in more complex cases, abdominal wall reconstruction (11, 12).

Complete mesh explantation is therefore generally considered the most reliable treatment for established mesh infection, although the extent of removal remains a matter of debate. Partial mesh removal may be considered when the non-infected portion of the prosthesis is well integrated and its complete removal would result in substantial loss of abdominal wall tissue or compromise subsequent reconstruction (13, 14). Nevertheless, preservation of any part of an infected prosthesis carries a risk of persistent or recurrent infection, and such an approach should therefore be reserved for carefully selected patients (15).

Available comparative data favor complete over partial mesh explantation when technically feasible. Kao et al. reported higher complication and reoperation rates following partial compared with complete mesh removal, while Gachabayov et al. observed a high rate of persistent infection after partial explantation (16, 17). Taken together, these findings suggest that partial mesh removal may have a role in selected circumstances, but complete explantation provides more reliable eradication of established infection.

Following removal of an infected mesh, the surgeon must balance two competing objectives: eradication of infection and restoration of abdominal wall integrity. Immediate implantation of a new permanent synthetic prosthesis is generally avoided in the setting of active infection because of the risk of recurrent prosthetic infection. Reconstructive options therefore include biological mesh reinforcement or autologous tissue repair without mesh. Biological mesh may provide additional reinforcement and reduce the risk of recurrence compared with mesh-free reconstruction, although reported recurrence rates remain substantial, ranging from approximately 18%–35% (18–20). Its use is also limited by cost and availability, and implantation of any prosthetic material into a previously infected field may carry a risk of further infectious complications (21, 22).

Autologous tissue reconstruction avoids implantation of additional foreign material and may therefore be particularly attractive in severely infected operative fields. Its principal limitation is the ability to achieve tension-free fascial closure, especially in patients with large abdominal wall defects. In such cases, component separation techniques can provide substantial medial advancement of the abdominal wall musculature and facilitate primary midline fascial closure while avoiding a bridged repair (23, 24). Although mesh-free reconstruction may carry a higher risk of subsequent hernia recurrence, any recurrence can potentially be addressed later under elective, non-infected conditions.

Most published experience with abdominal wall reconstruction following infected mesh explantation has involved biological meshes. Joliat et al. reported one of the larger recent series of patients undergoing reconstruction after removal of infected abdominal wall mesh (25). Most of the previously implanted prostheses in their series were located intraperitoneally, and all infected meshes were completely removed. Reconstruction was generally performed using a long-term absorbable biological mesh, most commonly placed in the retromuscular plane. Their results demonstrated that definitive single-stage reconstruction is feasible in many patients, while staged reconstruction with temporary negative-pressure wound therapy may be required when local infection precludes immediate reconstruction. Although hernia recurrence was relatively uncommon during follow-up, postoperative morbidity and the need for reoperation remained considerable. The main clinical message from this experience is therefore that complete mesh explantation followed by biological mesh reconstruction can provide effective abdominal wall restoration, but treatment remains complex and associated with substantial morbidity.

Our approach shares with Joliat et al. the principle that complete removal of infected prosthetic material is central to successful treatment. However, whereas their reconstructive strategy relied predominantly on immediate biological mesh implantation, our institutional preference is to minimize the introduction of additional foreign material in the presence of severe active infection whenever tension-free autologous fascial closure can be achieved.

In our patient, two factors were particularly important when planning surgical treatment. First, the patient had a longstanding and severe chronic mesh infection characterized by multiple high-output purulent fistulas and extensive local contamination. Second, a large recurrent ventral hernia was present and required simultaneous restoration of abdominal wall continuity. Our primary objective was therefore complete mesh explantation and radical debridement of all infected and devitalized tissue, followed, if technically possible, by definitive reconstruction using autologous tissue.

Given the size of the fascial defect, tension-free midline closure could not be achieved without additional abdominal wall mobilization. Anterior component separation was therefore performed because of its ability to provide substantial medial advancement of the rectus muscles and facilitate primary fascial closure. Biological mesh implantation was considered as a contingency option and would have been used only if primary closure remained impossible despite bilateral component separation.

The decision to avoid immediate biological mesh implantation was primarily influenced by the severity of the local infection. Multiple purulent cavities and extensive devitalized tissue were present at surgery. Although biological meshes can be used in contaminated or infected surgical fields, our preference in the presence of severe active infection is to avoid introducing additional prosthetic material when satisfactory primary fascial closure can be achieved. In this patient, we considered that complete eradication of infection took priority over reinforcement of the repair with a new implant.

A second consideration was preservation of future reconstructive options. Although biological mesh reinforcement may decrease the risk of early recurrence compared with mesh-free closure, recurrence rates of up to 35% have been reported. Placement of a biological mesh in the retromuscular plane would also occupy the preferred anatomical plane for any future definitive mesh-based reconstruction. We therefore considered complete infection eradication combined with mesh-free fascial closure to be the most appropriate initial strategy. Should a recurrent hernia develop, elective retromuscular mesh reconstruction could subsequently be performed under controlled, non-infected conditions.

At the latest follow-up, 16 months after surgery, the patient remained free of both recurrent infection and clinically evident hernia recurrence. Nevertheless, this follow-up period is still relatively short for assessment of long-term abdominal wall durability, and continued surveillance is warranted.

In addition to appropriate treatment of established mesh infection, prevention remains of fundamental importance. Several technical factors influence the risk of mesh-related complications, with mesh position being among the most important. Retromuscular (sublay) placement is generally regarded as the preferred anatomical position because it provides effective mechanical reinforcement while positioning the prosthesis within a well-vascularized tissue plane that is relatively protected from both superficial and intra-abdominal contamination. Available evidence consistently demonstrates more favorable surgical-site and recurrence outcomes for retromuscular repair compared with onlay, inlay, bridging, and intraperitoneal techniques (22, 25–28). Thus, whenever anatomical and clinical circumstances permit, retromuscular placement should be preferred for definitive ventral hernia reconstruction.

Achieving primary fascial closure is similarly important. Bridged repairs are associated with increased rates of surgical-site complications, mesh-related morbidity, and hernia recurrence. When tension-free midline closure cannot otherwise be achieved, component separation provides additional medial advancement and may avoid the need for bridging. When an anterior component separation is performed, preservation of periumbilical perforating vessels should be considered whenever technically possible, as perforator-sparing approaches reduce skin ischemia and wound morbidity and may consequently decrease the risk of subsequent prosthetic infection (29, 30).

Meticulous management of the subcutaneous tissues represents another important preventive measure. Elimination of dead space, careful hemostasis, and appropriate use of closed-suction drainage may reduce postoperative seroma and hematoma formation, thereby decreasing conditions that favor surgical-site infection and secondary mesh contamination.

Particular caution is required when considering permanent synthetic mesh implantation during emergency abdominal surgery. Emergency operations are frequently performed in patients with physiological derangement, hemorrhage, hypovolemia, systemic inflammatory responses, or inadequate opportunity for preoperative optimization. These factors may impair wound healing and host defense mechanisms and thereby increase susceptibility to surgical-site and prosthetic infection (31). The decision to implant permanent mesh in an emergency setting should therefore be individualized according to the indication for mesh placement, the degree of contamination, the patient's physiological condition, and the possibility of alternative reconstructive strategies.

The present case illustrates the potentially severe consequences that may follow implantation of a permanent intraperitoneal synthetic prosthesis under unfavorable emergency conditions. In this patient, a composite mesh was implanted during emergency splenectomy for traumatic splenic rupture, and chronic mesh infection subsequently developed, ultimately requiring complete explantation and complex abdominal wall reconstruction. Although the precise rationale for mesh implantation during the initial procedure remains unclear, this case emphasizes the importance of carefully considering whether permanent prosthetic reinforcement is truly necessary during an emergency operation.

When primary fascial closure cannot be safely achieved in an emergency setting, alternative approaches may be preferable to immediate placement of a permanent synthetic prosthesis, particularly when contamination is present or anticipated. Depending on the clinical circumstances, these alternatives may include temporary abdominal closure, selected use of biological or other non-permanent reconstructive materials, or a planned ventral hernia strategy with delayed definitive abdominal wall reconstruction after recovery from the acute event.

The degree of operative field contamination should therefore remain a central consideration when selecting a reconstructive technique. Permanent non-absorbable synthetic mesh should generally be avoided in actively infected fields, while its use in contaminated settings requires careful patient and procedure selection. In selected patients in whom prosthetic reinforcement is necessary despite contamination, biological meshes may represent one of the available reconstructive options (32).

Finally, it is important to distinguish composite meshes from biological prostheses. Composite meshes remain permanent synthetic implants despite incorporation of an anti-adhesive visceral barrier. These coatings are designed primarily to reduce adhesion formation between the prosthesis and intra-abdominal viscera and should not be interpreted as providing protection against bacterial colonization or mesh infection. Consequently, composite meshes should not be considered equivalent to biological materials or regarded as intrinsically suitable for implantation in actively infected operative fields (33).

## Conclusion

Mesh infection remains an important complication of ventral hernia repair and is likely to continue to be encountered in clinical practice. Its management is often complex and may require prolonged treatment and complex abdominal wall reconstruction. Nevertheless, the substantial reduction in hernia recurrence achieved with mesh-based repair has established mesh implantation as the standard of care for most ventral hernias, with its benefits generally outweighing the associated risks.

The present case illustrates the potential consequences of implanting a permanent intraperitoneal mesh during emergency abdominal surgery. In accordance with the principles of damage control surgery, implantation of a permanent synthetic mesh in a contaminated or physiologically compromised setting should be approached with caution, as it may predispose patients to severe infectious complications, as demonstrated in this case.

Future efforts should focus on preventing mesh infection through structured surgeon education on appropriate mesh selection, optimal mesh placement, and meticulous surgical technique. In addition, further refinement of evidence-based guidelines regarding the choice of mesh, its indications, and the optimal plane of implantation in different clinical settings may help reduce the incidence of mesh-related complications.

## Data Availability

The original contributions presented in the study are included in the article/supplementary material, further inquiries can be directed to the corresponding author.
